# Knockdown of two Cadherin genes confers resistance to Cry2A and Cry1C in *Chilo suppressalis*

**DOI:** 10.1038/s41598-017-05110-9

**Published:** 2017-07-20

**Authors:** Zan Zhang, Xiaolu Teng, Weihua Ma, Fei Li

**Affiliations:** 1 Hubei Insect Resources Utilization and Sustainable Pest Management Key Laboratory, College of Plant Science and Technology, Huazhong Agricultural University, Wuhan, 430070 China; 2 Department of Entomology, Nanjing Agricultural University, Nanjing, 210095 China; 3 Ministry of Agriculture Key Lab of Agricultural Entomology and Institute of Insect Sciences, Zhejiang University, 866 Yuhangtang Road, Hangzhou, 310058 China

## Abstract

*Bacillus thuringiensis* (Bt) Cry toxins play an important role in the management of insect pests. Resistance to Bt toxins has been reported in many pest insects but the mechanism responsible for this resistance in rice crop pests remains largely unknown. Cadherin is one of several Bt toxin receptors. At present, only one cadherin gene, *CsCAD1*, has been documented in the striped rice stem borer, *Chilo suppressalis*. We amplified a nearly full-length transcript of another *C. suppressalis* cadherin gene, *CsCAD2*, and found that it has a different expression pattern to *CsCAD1*. *CsCAD1* was highly expressed in fifth and sixth instar larvae, especially in the midgut, while the expression levels of *CsCA2* were equably in each developmental stage. Newly hatched larvae were fed on rice smeared with synthesized siRNA to knockdown either *CsCAD1* or *CsCAD2*, and then were fed transgenic rice expressing either the *Cry2A* or *Cry1C* toxins. The siRNA-treatment groups had lower mortality and higher survival rates than the control group, suggesting that reduced expression of *CsCAD1* or *CsCAD2* increased resistance to *Cry2A* and *Cry1C*. We conclude that *CsCAD1* and *CsCAD2* interact with Bt toxins in *C. suppressalis* and that this interaction could be the mechanism underlying Bt resistance in this insect.

## Introduction

Insecticidal Cry proteins from *Bacillus thuringiensis* (Bt) have been widely used to develop transgenic crops that have become an important part of agricultural pest management^1–3^. Cry toxins are ingested by digestive proteases in the midgut of insects where the activated toxins interact with midgut brush border membrane proteins, including cadherin^4^, ABC type C transporters (ABCCs)^5, 6^, alkaline phosphatase (ALP)^7, 8^, and aminopeptidase N (APN)^8, 9^. Cry toxins are integrated into the membrane, leading to pore formation, cell lysis and insect death^10^. However, the development of resistance to Bt toxins in many pest insects threatens to make transgenic Bt crops redundant^11^.

The development of resistance to Bt toxins in insects has been associated with mutation, down-regulation, or deletion, of Bt receptors^2, 12, 13^. Cadherin, a calcium-dependent cell adhesion protein^14^, is thought to be one of several such receptor proteins that bind to Cry toxins^15, 16^. The first such cadherin protein to be identified was the *Cry1A* toxin-binding protein in *Manduca sexta* ^17^, which was then found to be involved in binding Cry toxins in other Lepidopteran, coleopteran and dipteran insects^4, 18–23^. However, the affinity of cadherins for different Cry toxins varies in different insects. Fox example, some Cry toxins are not lethal to the Coleoptera or Lepidoptera^24, 25^.

The striped rice stem borer, *Chilo suppressalis* Walker, is one of the most destructive rice pests in China and other Asian countries. Transgenic rice strains expressing the Cry toxins *Cry2A* and *Cry1C* have been developed to protect rice crops from this notorious pest. However, it is likely that *C. suppressalis* will develop resistance to these toxins once transgenic rice becomes more widely grown. It is, therefore, important to understand the mechanisms that confer resistance to Cry toxins in this species.

A cadherin-like *C. suppressalis* gene (*CsCAD1*, AY118272) has been deposited in the NCBI GenBank^26–28^. We used Rapid-amplification of cDNA ends (RACE) to clone another *C. suppressalis* cadherin gene, which we named *CsCAD2*, and investigated the expression of both genes in different *C. suppressalis* developmental stages and tissues. We found that knockdown of these genes reduced sensitivity to both *Cry2A* and *Cry1C* in *C. suppressalis*.

## Results

### Amplifying the *CsCAD2* gene

We obtained a fragment of a cadherin gene from the transcriptome of *C. suppressalis* and, after mapping this fragment onto the *C. suppressalis* genome^29^, found that this gene had not previously been reported. We named this newly discovered gene *CsCAD2*. By amplifying the transcript with RACE and incorporating information from the *C. suppressalis* genome, we obtained a nearly full-length transcript of *CsCAD2*, including a 5′ untranslated coding region (UTR), open reading frame (ORF). The *CsCAD2* ORF was 4,912 bp, encoding 1,493 amino acids. The *CsCAD2* protein sequence had high identity with other insect cadherins, for example 88% with that of *Bombyx mori* and 86% with that of *Plutella xylostella*.

### Gene structure and phylogenetic analysis of *CsCAD1* and *CsCAD2*

**Figure 1:**
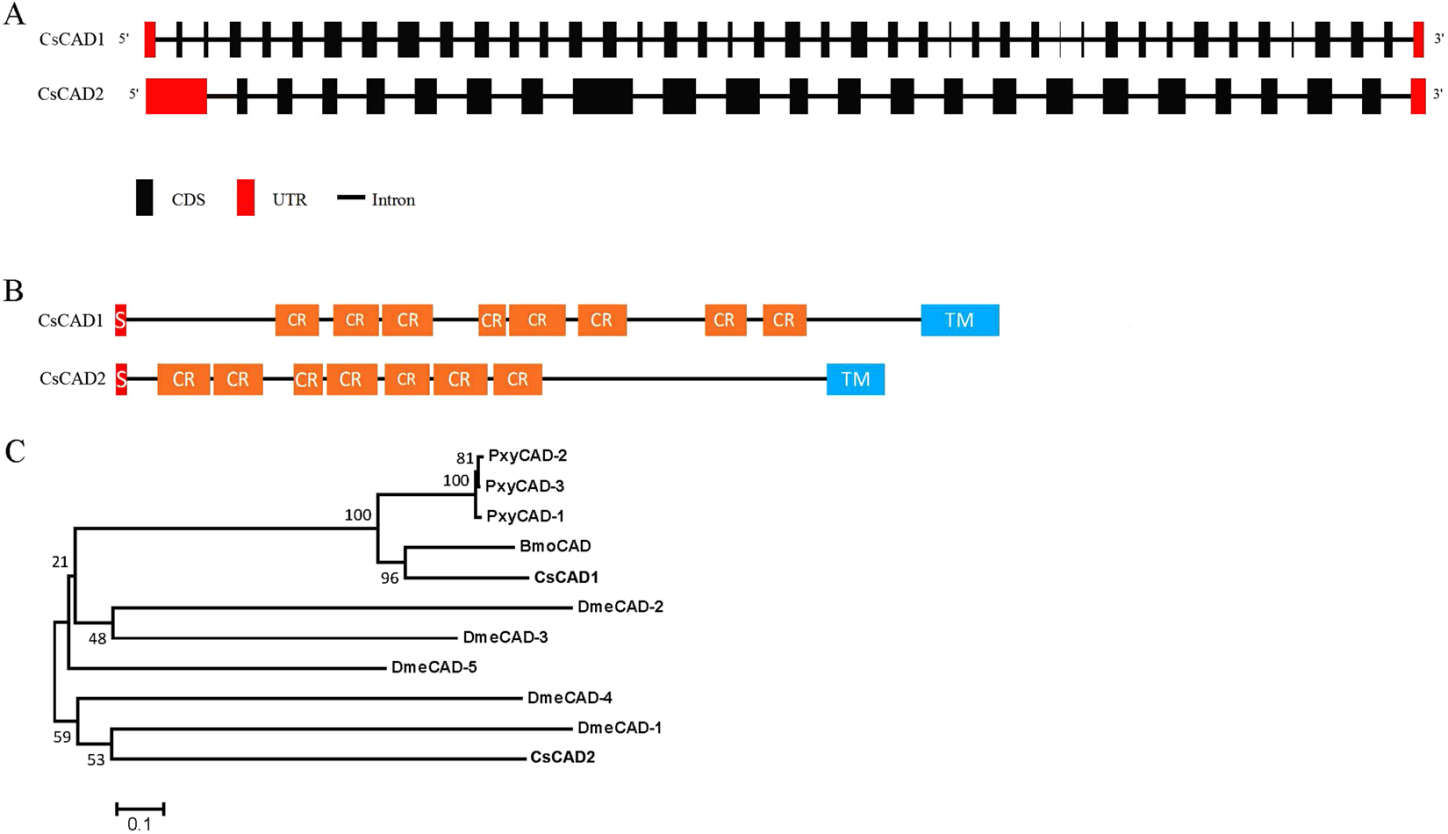
Structure (**A**), predicted domain structure (**B**) and phylogeny (**C**), of the *Chilo suppressalis* cadherin genes *CsCAD1* and *CsCAD2*. CR = characteristic cadherin repeat domains, S = signal peptide, TM = transmembrane domain. The phylogenetic tree of the relationships between *CsCAD1* and *CsCAD2* and eleven other Lepidopteran and Dipteran cadherin genes (**C**) was constructed using the neighbor-joining method in MEGA 6.0 with 1000 bootstrap replications. The GenBank accession numbers of the genes compared are AAM78590.1 and AGG36450.1 for *C. suppressalis* (*CsCAD1* and *CsCAD2*), NP_001036368.2, NP_650554.3, NP_731649.2, NP_788635.3 and NP_731930.2 for *Drosophila melanogaster* (*DmeCAD-1, DmeCAD-2, DmeCAD-3, DmeCAD-4* and *DmeCAD-5*), ADD92322, ABU41413.1 and NP_001036368.2 for *Plutella xylostella* (*PxyCAD-1, PxyCAD-2, and PxyCAD-3*), and NP_001037682.1 for *Bombyx mori* (*BmoCAD*).

The nucleic acid sequences of *CsCAD1* and *CsCAD2* were aligned with the genome of *C. suppressalis* to obtain the structures of both genes (Fig. 1A). The two genes are located in different scaffolds of the genome. *CsCAD1* had 41 exons and 40 introns and a length of 44,762 bp, whereas *CsCAD2* had only 24 exons and 23 introns and a length of 316,095 bp. Surprisingly, the first *CsCAD2* intron was very long; 253,600 bp. Conserved domain analysis indicates that both *CsCAD1* and *CsCAD2* have characteristics that are conserved in other cadherin proteins. *CsCAD1* had eight cadherin repeat domains, two Ca^2+^ binding sites and one trans-membrane region, whereas *CsCAD2* had seven cadherin repeat domains, five Ca^2+^ binding sites and one trans-membrane region (Fig. 1B). Comparison of *CsCAD1* and *CsCAD2* with nine other cadherin genes of three well-studied insects; *B. mori*, *P. xylostella* and *D. melanogaster*, indicated that *CsCAD1* was conserved in Lepidopteran. However, *CsCAD2* was not clustered with other Lepidopteran CADs but was adjacent with *Drosophila* CADs, which is worthy of further investigations (Fig. 1C).

### Temporal and spatial expression of *CsCAD1* and *CsCAD2*

**Figure 2:**
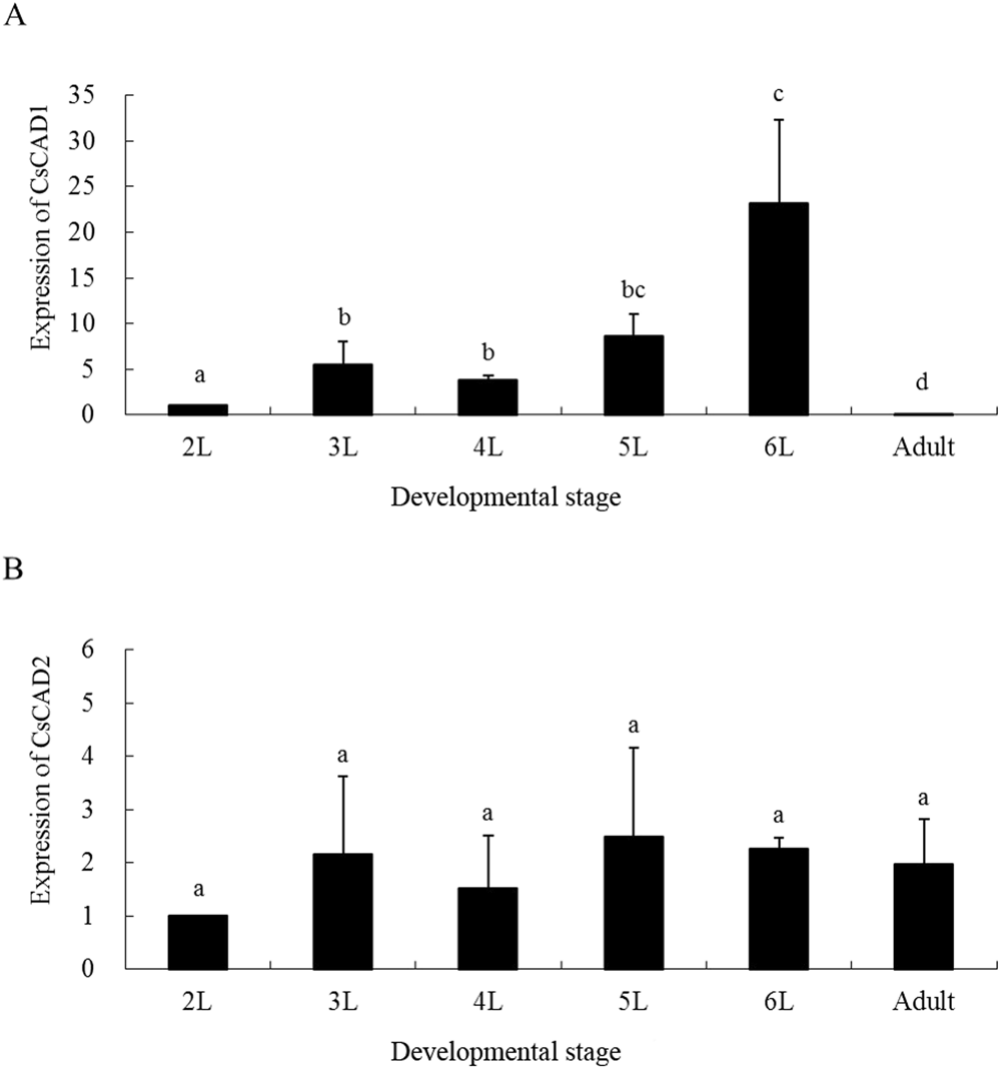
Relative abundance of mRNA of the *Chilo suppressalis* cadherin genes (**A**) *CsCAD1* and (**B**) *CsCAD2* in different larval instars and adults. Results are means ± SE. Bars with the same lowercase letter are not significantly different.

**Figure 3:**
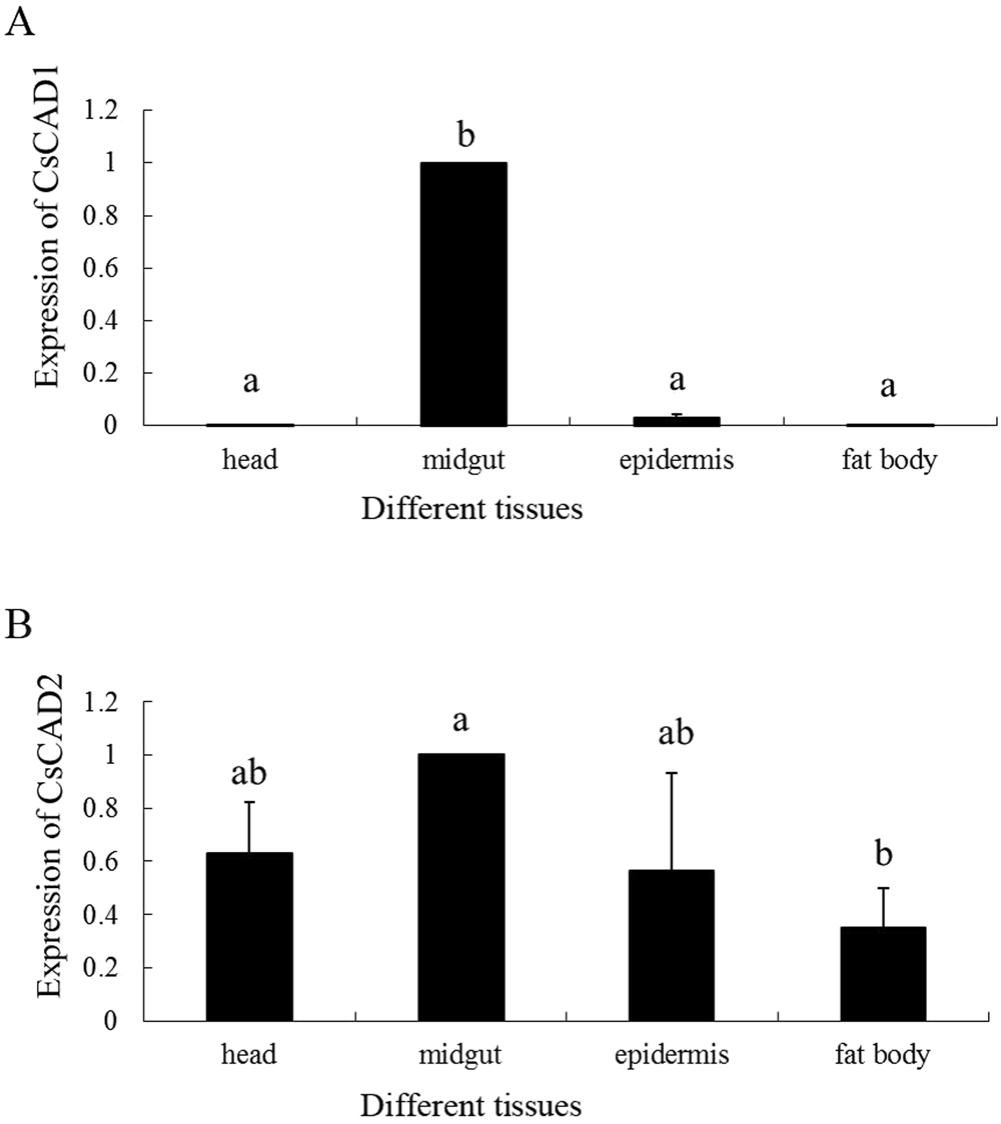
Relative abundance of mRNA of the *Chilo suppressalis* cadherin genes (**A**) *CsCAD1* and (**B**) *CsCAD2* in different body parts. Results are means ± SE. Bars with the same lowercase letter are not significantly different.

The expression patterns of both *CsCAD1* and *CsCAD2* were compared using quantitative PCR (qPCR). The house-keeping gene E2F and the G3PDH gene were used as internal controls. qPCR analyses of the expression of *CsCAD1* and *CsCAD2* in first to sixth instar larvae, and in adults, indicated that *CsCAD1* expression peaked in 6^th^ instar larvae and decreased markedly in adults. In contrast, *CsCAD2* was expressed in all developmental stages without significant difference (Fig. 2). *CsCAD1* expression was almost exclusively confined to the midgut and was detected only at very low levels in other tissues, including the head, epidermis and fat body (Fig. 3A). In contrast, although *CsCAD2* expression was also highest in the midgut, it was also highly expressed in the head and epidermis. Furthermore, although *CsCAD2* expression was lowest in the fat body, its expression in that organ was nearly half that in the midgut (Fig. 3B). This indicates that although *CsCAD1* expression is largely confined to the midgut, *CsCAD2* was expressed in all tested tissues. This was consistent with phylogenetic analysis, indicating that *CsCAD1* was conserved in Lepidopteran insects, while *CsCAD2* was a more ancient protein and conserved in almost all insects.

### Knockdown of *CsCAD1* and *CsCAD2* by RNA interference

**Figure 4:**
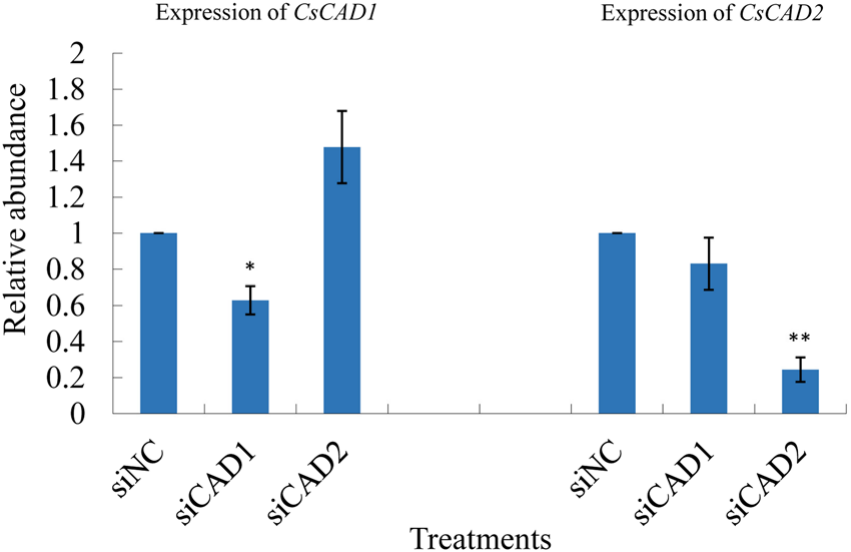
Relative expression of the *Chilo suppressalis* cadherin genes *CsCAD1* and *CsCAD2* 24 hrs after siRNA treatment. Results are means ± SE. Bars with the same lowercase letter are not significantly different; NC = negative control, si*CAD1* = *CsCAD1* knockdown treatment group, si*CAD2* = *CsCAD2* knockdown treatment group.

In order to investigate the functions of *CsCAD1* and *CsCAD2* we used siRNAs to silence both genes and a random sequence siRNA without any targets in *C. suppressalis* as a negative control (siNC). Newly hatched larvae were put into petri dishes containing rice stems smeared with siRNAs and the expression levels of *CsCAD1* and *CsCAD2* were measured with qPCR after 48 hrs (Fig. 4). The relative abundance of target gene transcripts was 40% of that in the control group, indicating that both target genes were successfully downregulated. The expression of non-target homolog genes was not affected, indicating that RNAi knockdown of *CsCAD1* and *CsCAD2* was achieved without influencing non-target genes.

### Silencing *CsCAD1* and *CsCAD2* reduced sensitivity to Bt toxins

The RNAi-treated larvae were transferred to feed on one of three rice varieties; transgenic *Cry2A* rice, transgenic *Cry1C* rice, and non-transgenic Minghui 63 (negative control).

**Figure 5:**
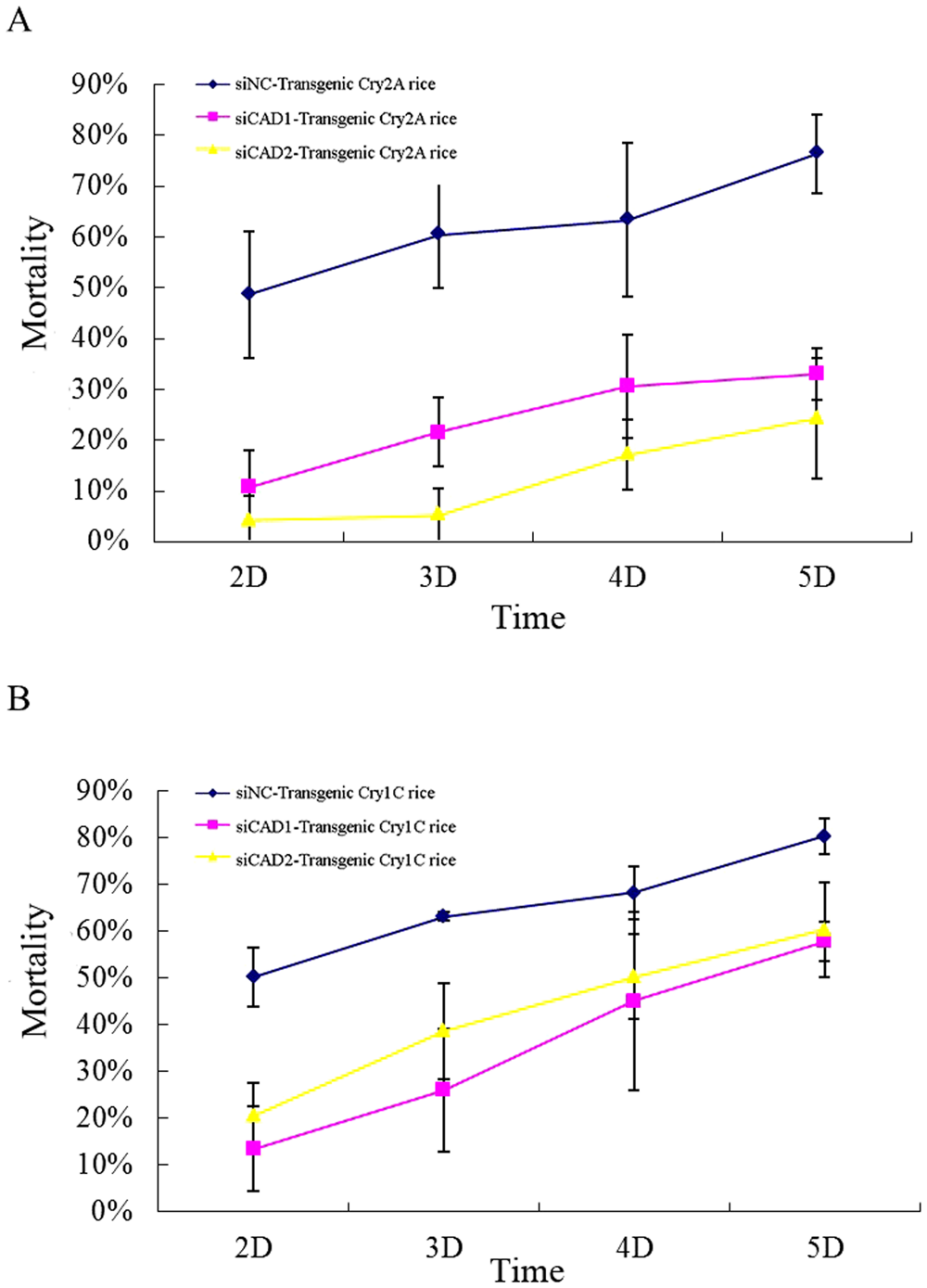
Mortality of *Chilo suppressalis* larvae treated with siRNA designed to silence the cadherin genes *CsCAD1* or *CsCAD2*, after feeding on transgenic rice expressing (**A**) *Cry2A* or (**B**) *Cry1C*; siNC = negative control, si*CAD1* = *CsCAD1* knockdown treatment group, siCAD2 = *CsCAD2* knockdown treatment group.

**Figure 6:**
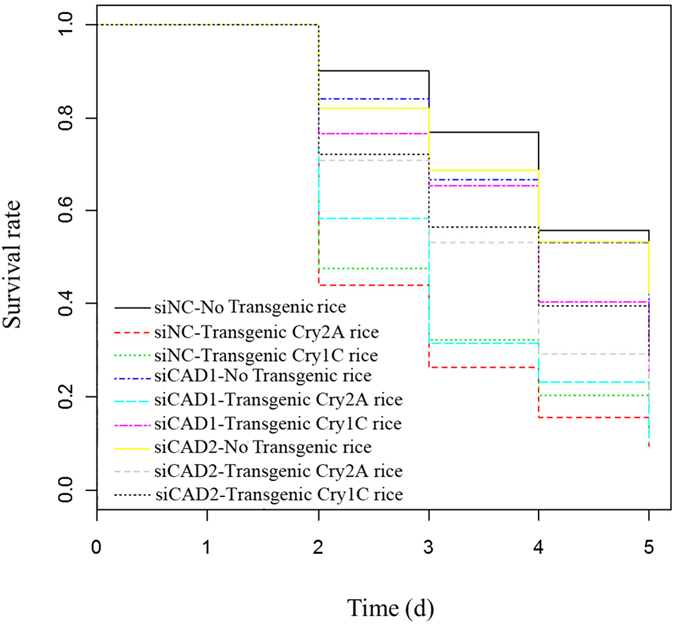
COX proportional hazard models for survival analysis; siNC-non-transgenic rice = negative control group feeding on non-transgenic rice, si*CAD1*-non-transgenic rice = the *CsCAD1* knockdown treatment group feeding on non-transgenic rice, si*CAD2*-non-transgenic rice = the *CsCAD2* knockdown treatment group feeding on non-transgenic rice, siNC-Transgenic Cry2A rice = the negative control group feeding on transgenic rice expressing Cry2A; si*CAD1*-Transgenic Cry2A rice = the *CsCAD1* knockdown group feeding on rice expressing Cry2A; si*CAD2*-Transgenic Cry2A rice = the *CsCAD2* knockdown treatment group feeding on transgenic rice expressing Cry2A, siNC-Transgenic Cry1C rice = negative control group feeding on transgenic rice expressing Cry1C, si*CAD1*-Transgenic Cry1C rice = the *CsCAD1* knockdown treatment group feeding on transgenic rice expressing Cry1C, si*CAD2*-Transgenic Cry1C rice = *CsCAD2* knockdown treatment group feeding on transgenic rice expressing Cry1C.

The death rate of the siNC treatment group was about 50% after feeding on transgenic *Cry2A* rice two days. In contrast, the mortality of the si*CAD1* treatment group was just 10%, and that of si*CAD2* treatment group only 5%, after two days (p < 0.05, Tukey’s HSD). Over the following three days, significant differences in mortality were observed between the two siRNA treatment groups and the siNC group. Over 70% of the siNC group were dead after 5 days whereas <30% of both siRNA treatment groups died over the same time period (Figs 5A and 6).

Similar results were obtained in experiments in which larvae were fed transgenic rice expressing the *Cry1C* toxin. In this case mortality in the si*CAD1* and si*CAD2* treatment groups were 10% and 20%, respectively, significantly lower than in the NC group (p < 0.05, Tukey’s HSD) (Figs 5B and 6). Interestingly, knockdown of *CsCAD2* conferred higher resistance to the *Cry2A* toxin than knockdown of *CsCAD1* (Fig. 5A). Conversely, silencing *CsCAD1* conferred higher resistance to the *Cry1C* toxin than to *CsCAD2* (Fig. 5B). This suggests that these two cadherin genes have different binding affinities for different Bt toxins.

## Discussion

Phylogenetic analysis of a newly cloned *C. suppressalis* cadherin gene, *CsCAD2*, suggests that this is most closely related to *D. melanogaster* cadherin genes. In contrast to *CsCAD1*, which was almost exclusively expressed in the midgut, *CsCAD2* were highly expressed in a number of different organs. This suggests that these two genes have different functions.

Recent studies have confirmed that *Cry1A* toxins interact with at least one type of receptor in the midgut^30, 31^. After the *Cry1A* toxin has been activated by enzymes in the midgut, it binds to the first receptor (midgut cadherin) with high affinity, which facilitates oligomerization of the toxins via a proteolytic process resulting in cell lysis. Previous studies have already indicated that cadherin is a receptor of Cry toxins in *M. sexta*, *B. mori, Diatraea saccharalis* and *Heliothis virescens* ^4, 32^. However, most studies have focused on cadherins that are specifically expressed in the midgut. Our results demonstrate that *CsCAD2* is both expressed in organs other than the midgut, and that knockdown of this gene increased resistance to both *Cry1C* and *Cry2A*. This suggests that this widely expressed gene is also probably a Bt toxin receptor.

We found that silencing either *CsCAD1* or *CsCAD2* reduced the sensitivity of *C. suppressalis* to Bt toxins, suggesting that both genes interact with Bt toxins in *C. suppressalis*. However, their differential expression in different tissues suggests that they could have different functions. This hypothesis is supported by the results of the RNAi experiment. The mortality of the si*CAD1* treatment group on transgenic *Cry2A* rice was slightly higher than that of the si*CAD2* treatment group, suggesting that *CsCAD2* has higher affinity for *Cry2A* than *CsCAD1*. However, when larvae were fed on transgenic *Cry1C* rice the mortality of the si*CAD2* treatment group was higher than that of the si*CAD1* treatment group, suggesting that the affinity of *CsCAD1* for *Cry1C* was higher than that of *CsCAD2*.

## Materials and Methods

### Insects and rice

*C. suppressalis* larvae of a strain susceptible to Bt toxins were collected in Wenzhou, Zhejiang province, China. Larvae were raised on rice seedlings in a laboratory at 25 ± 1 °C, 16/8 h light/dark and >80% humidity. The midguts of fourth instar larvae were dissected, immediately frozen in liquid nitrogen and stored at −70 °C until required for RACE.

Two transgenic rice strains, one expressing *Cry2A* and the other *Cry1C*, were used in experiments. These strains were derived from the same parental strain, Minghui 63, which was used as the negative control. Rice seeds were soaked for three days and then germinated in petri dishes on wet filter paper. All rice seeds were kindly provided by Prof. Yong-Jun Lin of Huazhong Agricultural University.

### Total mRNA isolation and cDNA synthesis

Whole bodies of different developmental stages of *C. suppressalis* (2^nd^ to 6^th^ instar larvae and adults), and specific body parts (head, midgut, epidermis, fat body), were first homogenized in a tissue grinder. TRIzol reagent (GIBCO, USA) was then used to isolate total mRNA from these samples according to the manufacturer’s protocol. Genomic DNA was removed from total RNA with a DNA-free kit (Ambion, USA). The integrity of the RNA obtained was checked by electrophoresis on a 1.5% agarose gel. The 260/280 nm absorbance ratios of all RNA samples were between 1.8 and 2.2. First strand cDNA was synthesized using M-MLV reverse transcriptase (Takara, Japan) with Oligo (dT18) as the anchor primer. The reaction mixtures were incubated at 70 °C for 10 min followed by 42 °C for one hour and 70 °C for 15 min. The cDNA was stored at −20 °C for further use.

### Quantitative real-time PCR

Quantitative real-time PCR (qPCR) was carried out with a SYBR Premix Ex Taq kit (Takara) using an ABI Prism 7300 (Applied Biosystems, USA) to detect the expression of *CsCAD1* and *CsCAD2* in the midgut of different development stages, and in different body parts. Primers were designed with Beacon Designer 7 (Table S1) and dissolution curves and gel electrophoresis were used to determine primer specificity. The amplification efficiency of all primers was checked with a cDNA dilution gradient, after which 2 μL of cDNA template was used in the PCR reaction according to the PCR kit’s protocol. The qPCR began at 95 °C for 30 secs, followed by 40 cycles of 95 °C for 5 secs, annealing at 60 °C for 31 sec, ending with cycles of 95 °C for 15 sec, 60 °C for 60 sec, and 95 °C for 15 sec. The specificity of the qPCR reactions was monitored with melting curve analysis using SDS software (version 1.4) and gel electrophoresis. Amplification efficiencies were determined by a series of template dilutions. All experiments were repeated in triplicate. The raw Ct values were obtained using ABI 7300 SDS software (Version 1.4). The standard Delta-Delta-Ct method was used to analysis the qPCR data. The housekeeping genes E2F and GAPDH (GenBank No.: DQ311161.1 and AB262581.1) were used as the internal controls. Significant differences among multiple means were determined using Tukey’s HSD (P < 0.05).

### RACE amplification of *CsCAD2*

Total RNA was extracted from the midgut of the 3^rd^ instar larvae. RACE amplification was carried out with a SMARTer RACE cDNA Amplification Kit (Takara) according to the manufacturer’s protocol. Fragments of *CsCAD2* cDNA were obtained from the transcriptome data used in previous studies^33^. The primers CTCATTACCTCCCTCCCACTCGGCAG (5′ RACE) and TGACAATCCACCACATTTCACGCAGG (3′ RACE) were designed, based on the sequences obtained, to amplify the full-length of the *CsCAD2* gene. The end-to-end primers (5′ AAACTTAATAGGCTTACTCGTTCTACC and 3′ GCTGTTCCCTGTCAAATGTCAC) were designed to amplify the full length of the *CsCAD2* gene. PCR products were inserted into vector (Takara, Dalian, China) and sequenced by the Nanjing Genscript Company, China. The transcriptome and genome data^29^ were used to obtain the full-length transcript of *CsCAD2*. The resultant sequence was submitted to GenBank (Accession No. JQ747493).

### RNA interference of cadherins

Two types of siRNA, si*CAD1* and si*CAD2*, were used to silence the *CsCAD1* and *CsCAD2* genes, respectively, and a random sequence siRNA was included as a negative control (siNC) (Table S2). All siRNAs were synthesized by the GenePharma Company. Larvae were treated with siRNAs in petri dishes with wet filter paper on the bottom. The siRNA was smeared onto 4 cm-long sprouts of non-transgenic rice and about 1000 newly hatched larvae were then put in the petri dishes to feed on the treated rice sprouts. Rice sprouts were replaced every 4 hrs for three days. All experiments were conducted at 27 °C and were repeated in triplicate so that there about 3000 insects were used per treatment and nearly 10 thousand in total.

Larvae from each treatment were used to assess both susceptibility to Bt transgenic rice and investigate gene expression. Susceptibility to transgenic rice was assessed in a randomly selected group of 90 larvae. These were subdivided into three groups of 30 which were randomly assigned to feed on either transgenic rice expressing *Cry2A* toxin, transgenic rice expressing *Cry1C* toxin, or non-transgenic rice. For another part, to examine the gene expression change, 50 insects were randomly selected at the 2^nd^ day after the insects were fed on transgenic rice. All experiments were repeated in triplicate.

### Gene evolution and domain analysis

Phylogenetic analysis was conducted in MEGA (v6.0) using the Neighbor-joining method with 1000 bootstrap replications. Domain structures of candidate cadherin genes were analyzed using CD Search (https://www.ncbi.nlm.nih.gov/Structure/cdd/wrpsb.cgi). Signal peptides were predicted with SignalP 4.1 (http://www.cbs.dtu.dk/services/SignalP/) and transmembrane domains with TMpred (http://www.ch.embnet.org/software/TMPRED_form.html). Cox’ proportional hazard model implemented in Program R (R Core Team R version 3.2.3) was used to analyze changes in survival from the 2^nd^ to 5^th^ day after larvae had commenced feeding on transgenic rice plants.

## Electronic supplementary material

Dataset 1
