# Supplementary material for: Knockdown of two Cadherin genes confers resistance to Cry2A and Cry1C in Chilo suppressalis
Source: Sci Rep. 2017 Jul 20;7:5992. doi: 10.1038/s41598-017-05110-9 (PMC5519675; doi:10.1038/s41598-017-05110-9)
Supplement: Supplementary file 1 — Dataset 1 [file 41598_2017_5110_MOESM1_ESM.doc]

**Knockdown of two Cadherin genes confers resistance to Cry2A and Cry1C in *Chilo suppressalis***

Zan Zhang^1, *^, Xiaolu Teng^2^, Weihua Ma^1^, Fei Li^3, *^

*1. Hubei Insect Resources Utilization and Sustainable Pest Management Key Laboratory, College of Plant Science and Technology, Huazhong Agricultural University, Wuhan 430070, China*

*2. Department of Entomology, Nanjing Agricultural University, Nanjing 210095, China*

*3. Ministry of Agriculture Key Lab of Agricultural Entomology and Institute of Insect Sciences, Zhejiang University, 866 Yuhangtang Road, Hangzhou 310058, China*

* Corresponding authors, Dr. Zan Zhang, zhangzan@mail.hzau.edu.cn; Prof. Dr. Fei Li, lifei18@zju.edu.cn

**Supplemental tables**

Supplemental table 1 Primers for qRT-PCR

| Genes |  | Primers |
| --- | --- | --- |
| CAD1 | Sense | 5'GTTGAGTGGTGGTAGTATGTGTCC 3' |
|  | Anti-sense | 5'CGGCGAGTTGCTGCGTATTG3' |
| CAD2 | Sense | 5'GACGGAACTTCAGCAAACAAC3' |
|  | Anti-sense | 5'CTCGGAATTATCAGTGGCAATG3' |
| G3PDH | Sense | 5'GTTGTGCCTCACCAATTTGTCAG3' |
|  | Anti-sense | 5'GCCACCTTCAGCGATGTCG3' |
| E2F | Sense | 5'ATTGCTGTGTGATAAAGAAGAAC3' |
|  | Anti-sense | 5'AGAAGGTGGTGGACTCAAC3' |

Supplemental table 2 siRNA oligo used for RNAi

| Name | siRNA |
| --- | --- |
| siCAD2 | 5'CUGGGCCGAUUAGAUUAAATT3' |
|  | 5'UUUAAUCUAAUCGGCCCAGTT3' |
| siCAD1 | 5'GCCUUCAUCAUGAGUACAATT3' |
|  | 5'UUGUACUCAUGAUGAAGGCTT3' |
| siNC | 5'UUCUCCGAACGUGUCACGUTT3' |
|  | 5'ACGUGACACGUUCGGAGAATT3' |
